# Differences in preoperative planning for high-tibial osteotomy between the standing and supine positions

**DOI:** 10.1186/s43019-021-00090-7

**Published:** 2021-03-01

**Authors:** Takehiko Matsushita, Shu Watanabe, Daisuke Araki, Kanto Nagai, Yuichi Hoshino, Noriyuki Kanzaki, Tomoyuki Matsumoto, Takahiro Niikura, Ryosuke Kuroda

**Affiliations:** Department of Orthopedic Surgery, Kobe University Graduate School of Medicine, 7-5-1 Kusunoki-cho, Chuo-ku, Kobe, Hyogo 650-0017 Japan

**Keywords:** High-tibial osteotomy, Preoperative planning, Standing, Supine

## Abstract

**Introduction:**

Previous studies have reported that alignment changes depend on the patient’s position in orthopedic surgery. However, it has not yet been well examined how the patient’s position affects the preoperative planning in high-tibial osteotomy (HTO). Therefore, the aim of this study was to investigate the effects of the patient’s position on preoperative planning in HTO.

**Materials and methods:**

A total of 60 knees in 55 patients who underwent HTO were retrospectively examined. Virtual preoperative planning for medial open-wedge HTO (OWHTO), lateral closed-wedge HTO (CWHTO), and hybrid CWHTO were performed by setting the percentage of the weight-bearing line (%WBL) at 62% as an optimal alignment. The correction angle differences between the supine and standing radiographs were measured. The virtual %WBL (v%WBL) was determined by applying the correction angle obtained from the standing radiograph to the supine radiograph. The %WBL discrepancy (%WBLd) was calculated as v%WBL − 62 (%) to predict the possible correction errors during surgeries. A single regression analysis was performed to examine the correlation between the correction angle difference and %WBLd.

**Results:**

The mean correction angle was significantly higher when the preoperative planning was based on standing radiographs than when based on supine radiographs (*P* < 0.001), and the mean difference was 2.2 ± 1.5°. The difference between the two conditions in the medial opening gaps for OWHTO, lateral wedge sizes (mm) for CWHTO, and hybrid CWHTO were 2.6 ± 2.0, 2.3 ± 1.6, and 1.9 ± 1.4, respectively. The mean v%WBL was 71.2% ± 7.3%, and the mean %WBLd was 10.1% ± 7.4%. A single regression analysis revealed a linear correlation between the correction angle difference and %WBLd (%WBLd = 4.72 × correction angle difference + 0.08). No statistically significant difference in the parameters was found between the supine and standing radiographs postoperatively.

**Conclusions:**

We found significant differences in the estimated correction angles between the supine and standing radiographs in the planning for HTO. Therefore, surgeons should carefully consider the difference between supine and standing radiographs and estimate the possible correction error during surgery when planning a HTO.

## Introduction

High-tibial osteotomy (HTO) is widely performed for the treatment of medial compartmental knee osteoarthritis (OA) and in patients with a varus malalignment. The basic principle of HTO is to reduce the mechanical load on the medial compartment of the knee by shifting the weight-bearing line (WBL) to the lateral compartment [1]. It has been reported that acquiring a correct alignment is one of the critical points for obtaining successful long-term outcomes of HTO [2–5] and precise preoperative planning is one of the most important steps in HTO. Although no definite optimal alignment exists, the lateral one third (approximately 62.5%) of the tibial plateau is a well-accepted alignment after surgery, allowing for better cartilage regeneration with favorable clinical outcomes [1, 6–8].

Preoperative planning for HTO is generally performed on anteroposterior (AP) long-leg-view radiographs using several methods [9–11]. In addition, various preoperative planning methods using picture archiving and communication systems (PACS), digital planning software, and computed tomography for three-dimensional planning have also been reported [12–17]. Although these new planning techniques can improve the accuracy of the bony correction in HTO, the effects of soft-tissue laxity have not been previously addressed. Recently, the importance of assessing the soft-tissue laxity in HTO has been increasingly emphasized [18–21]. In addition, several previous studies have reported that the alignment changes depend on several conditions such as the supine position, double-leg standing, and single-leg standing [21–24]. However, it has not yet been reported how the position of the patient influences preoperative planning in HTO.

Therefore, the aim of this study was to investigate the effects of the patients’ position on preoperative planning using a PACS system in HTO. We hypothesized that a significant difference in preoperative planning exists between the supine and standing conditions, and that the discrepancy can cause significant correction error during surgery.

## Materials and methods

### Demographics

**Fig. 1:**
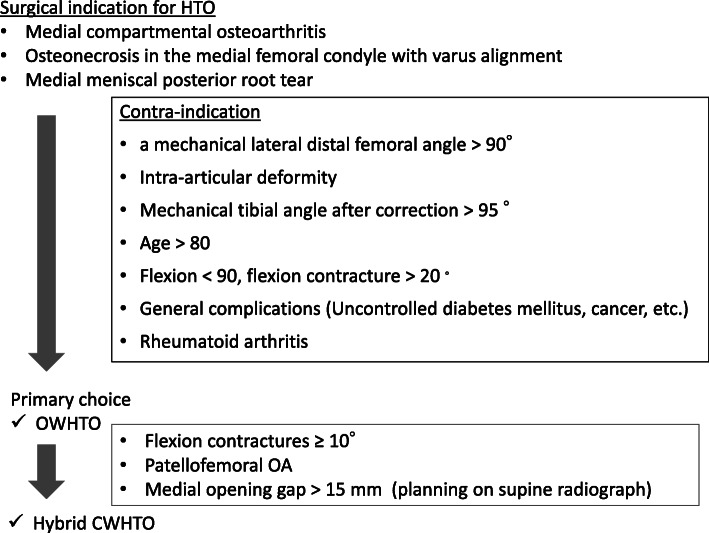
Summary of surgical indications and contraindications for high tibial osteotomy

The study protocol was reviewed and approved by the Institutional Review Board of our hospital (approval no. 170176). A total of 60 consecutive knees from 55 patients (male, 28; female, 27; mean age at the time of surgery, 59 ± 8.7 years; mean body mass index, 26.6 ± 5.6) who underwent medial open-wedge HTO (OWHTO) or hybrid lateral closed-wedge HTO (hybrid CWHTO) between 2016 August (when we started to use supine radiographs for preoperative evaluation) and 2019 March in our hospital were retrospectively examined (Table 1). All surgeries were performed by one surgeon (TM). The inclusion criteria were as follows: patients who received AP long-leg-view radiographs in the supine and standing positions preoperatively. Twelve knees in 10 patients who received HTO during that time were excluded from the study owing to the lack of radiographic data. The indications for HTO were symptomatic medial compartmental osteoarthritis and osteonecrosis with a varus malalignment (Fig. 1). Eleven patients received medial meniscal repair, four underwent osteochondral autograft transfer, and one received anterior cruciate ligament reconstruction in conjunction with HTO.

**Table 1:** Patient demogrphics

	Total	OWHTO	Hybrid CWHTO
Knees/patients	60/57	42/41	18/16
Age (years)	59 ± 8.7	62.5 ± 8.7	57.7 ± 8.4
Male/female	29/28	24/17	5/11
Height (cm)	162.0 ± 8.7	163.8 ± 9.0	157.8 ± 6.1
Weight (kg)	68.9 ± 14.7	71.1 ± 15.6	63.9 ± 11.4
BMI (kg/m^2^)	26.2 ± 4.4	26.6 ± 4.6	25.7 ± 3 .8

*BMI* body mass index, *CWHTO* closed-wedge high-tibial osteotomy, *OWHTO* open-wedge high-tibial osteotomy

### Radiographic limb-alignment analysis

**Fig. 2:**
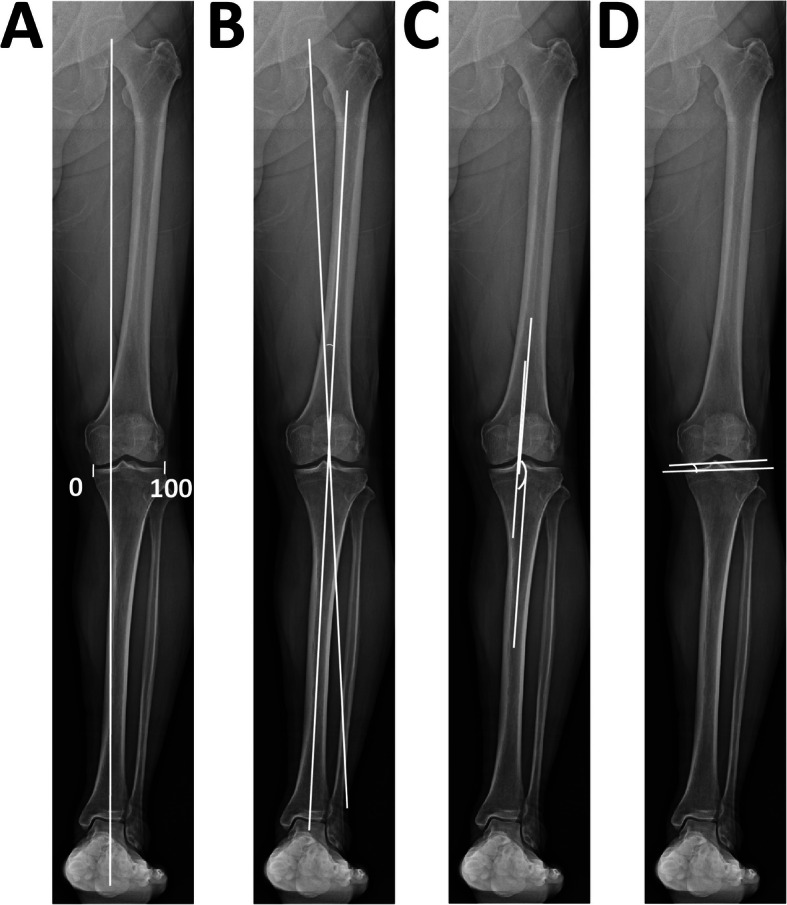
Radiographic analysis of limb alignment. *HKAA* hip-knee-ankle angle, *FTA* femorotibial angle, *WBL* % weight-bearing line, *JLCA* joint-line convergence angle

Plain AP long-leg-view radiographs were taken in the supine and double-leg standing positions to examine leg alignments preoperatively and 2 months after the surgery. The WBL was determined as the line from the center of the hip to the center of the ankle. The crossing point of the WBL at the tibial plateau was expressed as the percentage of the total length of the tibial plateau (%WBL), setting the most medial edge to 0% and the lateral edge at 100%. The %WBL was measured on standing and supine radiographs. The hip-knee-ankle angle (HKAA) was measured as the angle between the line from the hip center to the knee center and the line from the ankle center to the knee center. The varus alignment was expressed as a negative value, and the valgus, as a positive value. The femorotibial angle (FTA) was determined by measuring the angle between the center line of the distal one third of the femoral shaft and the center line of the proximal one third of the tibial shaft. The joint-line convergence angle (JLCA) was used to measure the angle between the line tangential to the medial and lateral condyles and the line parallel to the tibial joint surface (Fig. 2). The %WBL, HKAA, FTA, and JLCA were measured on standing and supine radiographs. All the measurements were performed using the PACS software (Shade Quest/View R-DG ver. 1.27, Fujifilm Solution Co., Ltd., Tokyo, Japan). For the angle and length data, the software expressed the data as numbers rounded to two decimals. The number rounded to one decimal was used in this study. The percentage of the length of the two lines was calculated as an integral number and the %WBL was also expressed as an integral number.

### Virtual preoperative planning

**Fig. 3:**
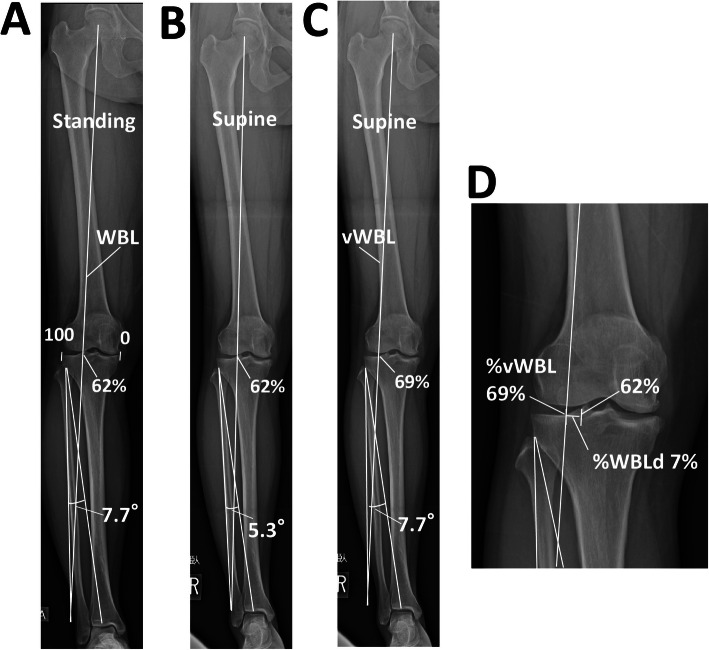
An example of preoperative planning for open-wedge high-tibial osteotomy. **a** Planning on a standing radiograph setting 62% as the target alignment. The estimated correction angle was 7.7°. **b** Planning on a supine radiograph. The estimated correction angle was 5.3°. **c** A virtual weight-bearing line (vWBL) was drawn by applying the correction angle (7.7°) determined on the standing radiograph to the supine radiograph. **d** The virtual %WBL (v%WBL) was 69%. The %WBL discrepancy (%WBLd) was calculated as 69–62 = 7 (%)

Preoperative alignment analysis and planning were performed according to Miniacci’s method on long-leg-view radiographs using the PACS, as previously reported [9, 12]. A total of 62.5% of the lateral plateau was set as the virtual optimal alignment because this value has been most widely used as the target alignment in HTO. As explained in the previous section, the percentage was calculated as an integral number in the PACS. Therefore, 62% was set as the virtual optimal alignment to simplify the measurement. Briefly, a line was drawn from the hip center to the ankle level through 62% of the lateral plateau as the optimal WBL. A line was drawn to the hinge point, and a line with the same length was drawn to reach the optimal line. The angle formed by the two lines was measured as the correction angle. For OWHTO, the lateral cortex of the tibia just next to the tip of the fibular head was chosen as the hinge point. A virtual starting point on the medial side of the tibia for transverse cutting was set as 3.8 to 4 cm distal from the medial joint space. The medial opening gap was measured on standing and supine radiographs (Fig. 3a).

**Fig. 4:**
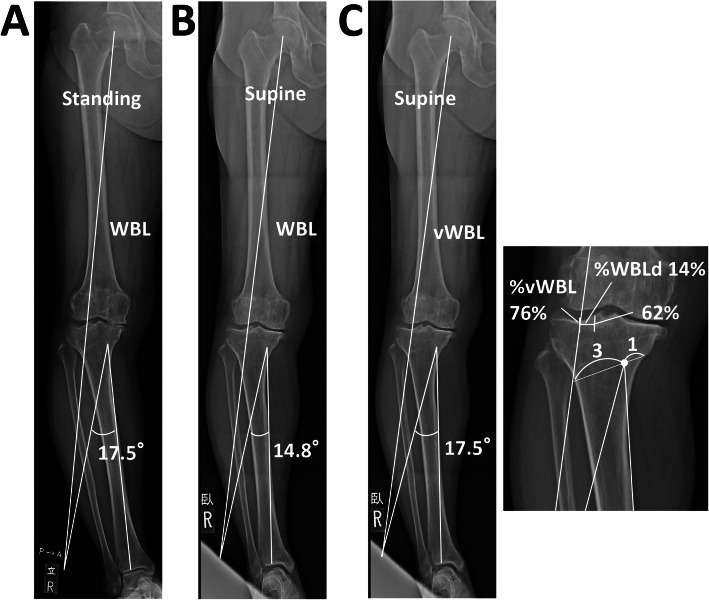
Example of preoperative planning for hybrid closed high-tibial osteotomy. **a** The hinge point was set as a point at a ratio of 1:3 during planning on the standing radiograph. **b** Planning on the supine radiograph. **c** A virtual weight-bearing line (vWBL) was drawn by applying the correction angle (17.5°) determined on the standing radiograph to the supine radiograph. **d** Virtual %WBL (v%WBL) was 76%. %WBL discrepancy (%WBLd) was calculated as 76–62 = 14 (%)

For CWHTO, the hinge point was set as the point 2 cm distal from the medial joint level, and a cutting line was set as the line connecting the point 4 cm distal to the lateral joint level and the point 2 cm distal from the medial joint level. For hybrid CWHTO, the hinge point was set as a point at a ratio of 1:3 from the medial point. The wedge size was determined according to the correction angle on standing and supine radiographs (Fig. 4a, b).

To examine possible correction errors occurring during the surgery, the %WBL discrepancy (%WBLd) was determined as follows: the correction angles to achieve 62%WBL were calculated using radiographs in the standing and supine positions according to the method described earlier. Then, the virtual %WBL (v%WBL) was determined by applying the correction angle obtained from the standing radiograph to the supine radiograph. The %WBLd was calculated as v%WBL (%) − 62% (Figs. 2c and 3c). All the three virtual plans for OWHTO, CWHTO, and hybrid CWHTO were performed for each patient, regardless of the actual surgical method.

### Surgical planning

In the actual planning of each surgery, the target alignments were determined according to the preoperative condition of the limb alignment and the osteoarthritic (OA) change. For relatively mild OA knees (%WBL: 20–40%, most areas of the medial compartment cartilage: International Cartilage Repair Society (ICRS) grades I–III [25]), the target alignment was set as 55–60%. For advanced OA knees (%WBL: < 20%, medial compartment cartilage: ICRS grade III or IV), the target alignment was set as 58–63% while limiting the postoperative medial proximal tibial angle (MTPA) within 95°).

### Surgical procedures and postoperative rehabilitation

For OWHTO, the medial proximal tibia was exposed using a straight incision, and the superficial medial collateral ligament was released. Biplane frontal and transverse cutting was performed using an oscillating bone saw and chisels. The osteotomy site was opened using an opener until the intended alignment had been reached. The gap distance between the most posteromedial cortex was measured using a caliper. Two wedge-shaped, β-tricalcium phosphate blocks (OSferion60, Olympus Terumo Biomaterials Corp., Tokyo, Japan), depending on the size of the gap, were placed into the gap. A medial locking compression plate (TomoFix, DePuy Synthes, Solothurn, Switzerland, or TriS plate, Olympus Terumo Biomaterials Corp., Tokyo, Japan) was used to fix the tibia.

Hybrid CWHTO was performed according to a previously reported method [26]. A 5-cm straight skin incision was made on the lateral side of the lower leg. The mid-shaft to the distal one third of the fibula was approached between the peroneus and gastrocnemius muscles, and an approximately 2-cm length of the fibula was resected. A 7-cm curved skin incision was made over the lateral proximal lower leg. The fascia of the tibialis anterior was incised, and the proximal lateral one third of the tibia was exposed. Osteotomy was performed as described in the section above. The wedge-shaped bone was resected depending on the angle and width determined on the preoperative supine radiograph. A locking plate was used to fix the tibia (TomoFix, DePuy Synthes, Solothurn, Switzerland, or TriS plate, Olympus Terumo Biomaterials Corp., Tokyo, Japan).

Partial weight-bearing was initiated 1 week after the surgery, and full weight-bearing was permitted at 4 weeks after the surgery.

### Statistical analyses

Based on preliminary results, a priori power analysis using G*Power (Heinrich Heine Universitȁt Dȕsseldorf, Germany) showed that a minimum of 52 patients were required to detect the difference in the correction angle between the two groups with a power of 0.95 and an *α* of 0.05. Student’s *t* test was used for comparisons between the two groups. Pearson’s correlation analysis was used to determine the correlation between the correction angle difference and the %WBLd, HKAA difference, JLCA difference, and FTA difference. Single linear regression analysis was performed to examine the presence of a linear correlation between the correction angle difference and %WBLd. A *P* value < 0.05 was set as statistically significant. Intraobserver and interobserver reliabilities were assessed using intraclass correlation coefficients (ICC). To examine the intraobserver reliability, measurements were performed at two different times with intervals of > 2 weeks. Interobserver reliability was examined by two independent observers. Intraobserver ICC for %WBL, HKA, JLCA, and FTA were 0.94, 0.92, 0.91, and 0.91, respectively. Interobserver ICC for %WBL, HKA, JLCA, and FTA were 0.93, 0.92, 0.91, and 0.90, respectively. All the tests were performed using SPSS for Windows version 16 (SPSS Inc., Chicago, IL, USA). The distribution graphs were created using Excel (Microsoft Corp., Redmond, WA, USA).

## Results

The mean %WBL, FTA, and JLCA were significantly higher in the standing radiographs than in the supine radiographs (*P* < 0.001), while the HKAA was significantly lower in the standing radiographs (*P* < 0.001; Table 2). In the preoperative virtual planning, the mean correction angle in the standing and supine radiographs was 11.6 ± 3.1° and 9.4 ± 2.7°, respectively, and the mean correction angle difference was 2.2 ± 1.5°. The mean medial opening and lateral closing gaps in the standing and supine positions in the preoperative planning for OWHTO, CWHTO, and hybrid CWHTO are shown in Table 3. The difference between the two conditions in terms of the medial opening gap for OWHTO, lateral wedge size for CWHTO, and the lateral wedge size for hybrid CWHTO were 2.6 ± 2.0 mm (range, 0–8.2 mm), 2.3 ± 1.6 mm (range, 0–6.5 mm), and 1.9 ± 1.4 mm (range, 0–4.8 mm), respectively (Table 3).

**Table 2:** Preoperative radiographic limb-alignment analyses

Preop.	Total			OWHTO			Hybrid CWHTO		
	Standing	Supine		Standing	Supine		Standing	Supine	
%WBL(%)	14.3 ± 14.2	23.4 ± 12.1	*P* < 0.01	19.0 ± 12.1	27.6 ± 10.0	*P* < 0.01	3.5 ± 13.0	13.7 ± 11.3	*P* < 0.01
HKAA (°)	−7.3 ± 3.4	−5.6 ± 3.0	*P* < 0.01	−5.9 ± 2.7	− 4.4 ± 2.3	*P* < 0.01	−10.5 ± 2.5	− 8.5 ± 2.3	*P* < 0.01
JLCA (°)	3.3 ± 1.9	1.9 ± 1.5	*P* < 0.01	2.8 ± 1.8	1.5 ± 1.4	*P < 0.01*	4.7 ± 1.7	3.0 ± 1.5	*P* = 0.01
FTA (°)	182.0 ± 3.1	180.6 ± 2.9	*P* < 0.01	181.2 ± 2.7	179.7 ± 2.6	*P* < 0.01	182.8 ± 2.6	183.3 ± 3.3	NS

*FTA* femorotibial angle*, JLCA* joint-line carrying angle*, NS* not significant, %*WBL* weight-bearing line

**Table 3:** Mean medial opening gap and lateral closing gap in standing and supine positions

	Standing	Supine	Difference
Medial opening gap (mm)	13.9 ± 4.0	11.3 ± 4.0	2.6 ± 2.0
Lateral closing gap (mm)			
Conventional	12.4 ± 3.5	10.2 ± 2.9	2.3 ± 1.6
Hybrid (1:3)	9.6 ± 2.8	7.8 ± 2.3	1.9 ± 1.4

**Fig. 5:**
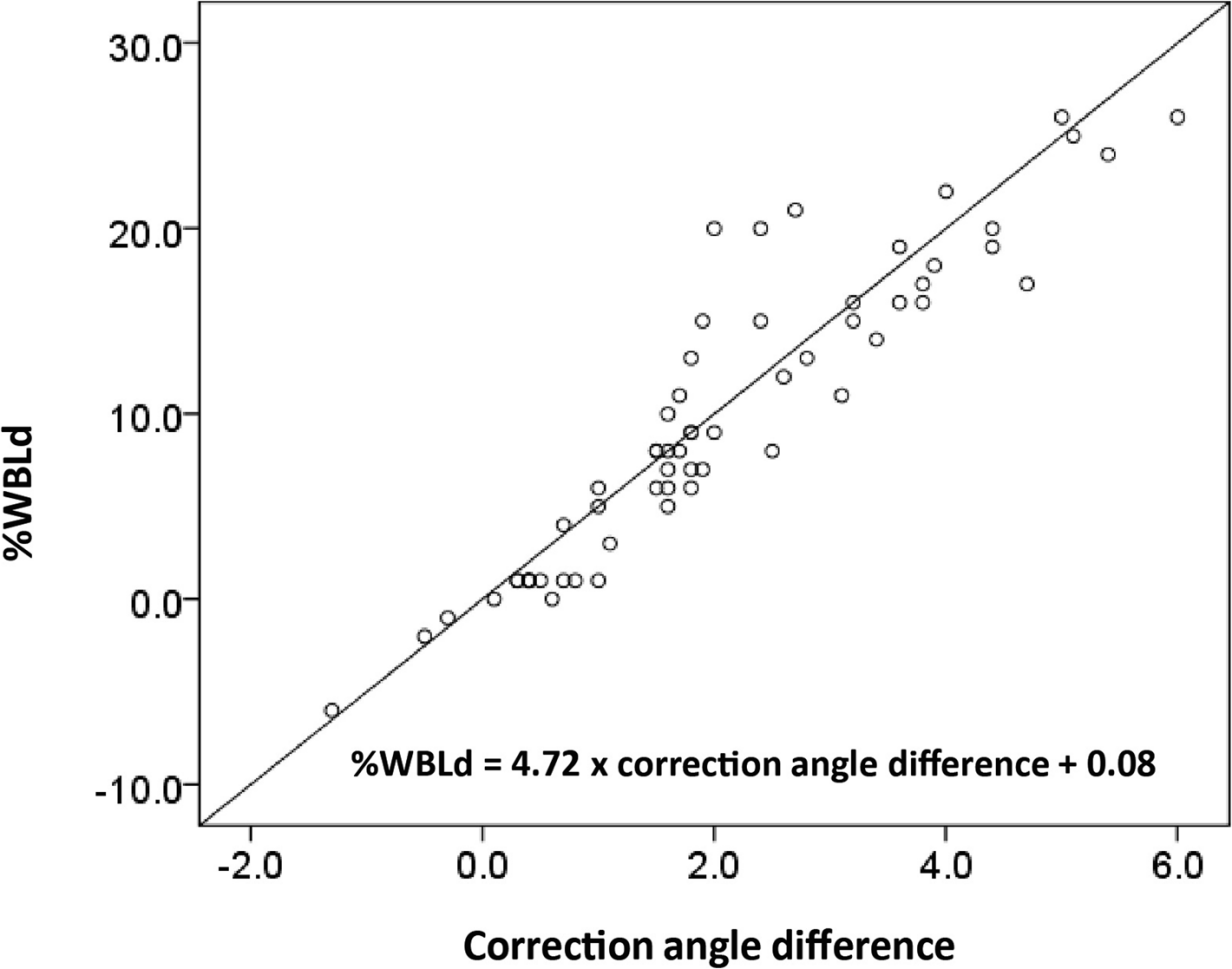
Linear regression analysis between the correction angle and weight-bearing line (%WBLd)

Significant correlations were found between the difference in correction angle and the differences in JLCA, FTA, HKAA, and %WBLd (Table 4). The mean v%WBL was 71.2 ± 7.3%, and the %WBLd was 10.1 ± 7.4%. The single regression analysis revealed a linear correlation between the correction angle difference and %WBLd (%WBLd = 4.72 × correction angle difference + 0.08; Fig. 5).

**Table 4:** Pearson’s correlation analyses for correlation between the correction angle difference and the %WBLd, HKAA difference, JLCA difference, and FTA difference

	Pearson’s correlation coefficent	
%WBLd	0.93	*P* < 0.01
HKAA difference	−0.50	*P* < 0.01
JLCA difference	0.34	*P* < 0.01
FTA difference	0.35	*P* < 0.01

*FTA* femorotibial angle, *JLCA* joint-line carrying angle, *HKAA* hip-knee-ankle angle, *%WBLd* weight-bearing line discrepancy

**Fig. 6:**
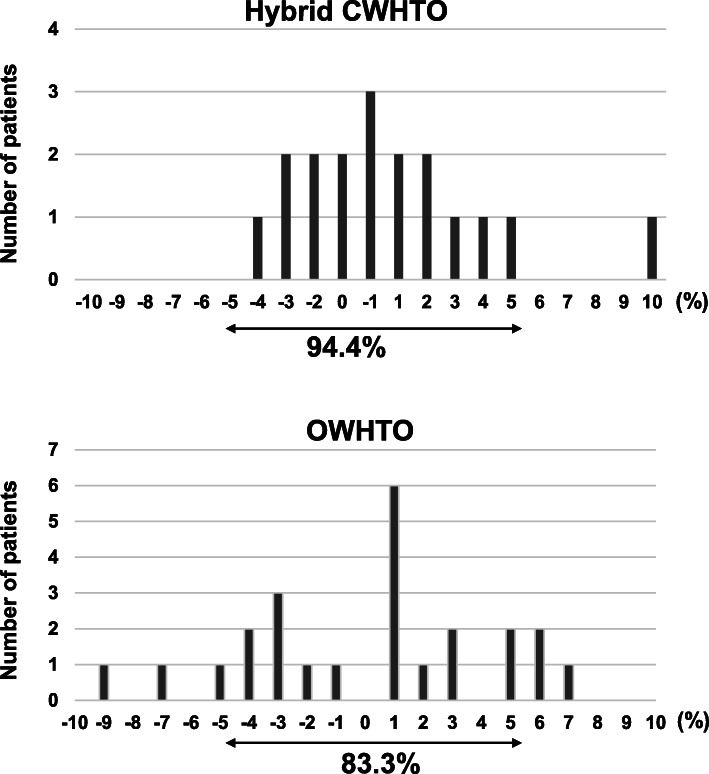
The distribution pattern of weight-bearing line (%WBL) difference between standing and supine radiographs after surgery. The vertical axis shows the number of patients and the horizontal axis shows %WBL difference (%WBL in standing position – %WBL in supine position)

In the actual postoperative alignment analyses, there were no statistically significant differences in the parameters between the supine and standing radiographs postoperatively (Table 5). All the examined parameters were significantly changed after the surgery as compared with before surgery on both supine and standing radiographs (all the *P* values for %WBL, HKAA, and FTA were < 0.01), except for the JLCA on supine radiographs. In the evaluation of %WBL difference between postoperative standing and supine radiographs, 94.4% of the patients were distributed within a 5% difference after hybrid CWHTO. In one patient, who had an overcorrection with a postoperative %WBL of 88% due to a technical error, showed a 10% increase in the standing position. In the patients who received OWHTO, 83.3% of the patients were within 5% (Fig. 6). There was no obvious tendency toward an increase or a decrease in %WBL between standing and supine radiographs after surgery.

**Table 5:** Postoperative radiographic limb-alignment analyses

Postoperative	Total			OWHTO			Hybrid CWHTO		
	Standing	Supine		Standing	Supine		Standing	Supine	
%WBL (%)	57.5 ± 10.0	57.6 ± 8.6	NS	57.0 ± 8.3	57.9 ± 7.2	NS	58.9 ± 13.5	58.0 ± 11.2	NS
HKAA (°)	1.7 ± 2.5	1.8 ± 2.2	NS	1.6 ± 2.2	1.8 ± 1.9	NS	1.8 ± 3.2	1.8 ± 2.8	NS
JLCA (°)	2.2 ± 1.5	1.9 ± 1.7	NS	2.0 ± 1.5	1,7 ± 1.8	NS	2.7 ± 1.5	2.6 ± 14	NS
FTA (°)	171.8 ± 2.6	171.9 ± 2.4	NS	171.9 ± 2.2	171.9 ± 2.0	NS	171.5 ± 3.5	171.9 ± 3.0	NS

*CWHTO* closed-wedge high-tibial osteotomy, *FTA* femorotibial angle, *JLCA* joint-line carrying angle, *HKA*, *NS* not significant, *OWHTO* open-wedge high-tibial osteotomy, *%WBL* weight-bearing line

## Discussion

The most significant finding of this study was that the estimated correction angle was significantly higher when standing radiographs were used during the planning than when supine radiographs were utilized, and the mean %WBL discrepancy was approximately 10%. In addition, a linear correlation between the correction angle difference and %WBL discrepancy was found between the standing and supine positions. Meanwhile, no statistically significant differences in the parameters were found between the supine and standing radiographs postoperatively.

Sabharwal et al. examined the difference in mechanical axis deviation between the standing whole-leg radiographs and supine fluoroscopic images of the lower extremity using an electrocautery cord in 102 limbs of 80 patients who underwent surgeries for osteotomies, epiphysiodesis, and removal of fixators. They reported that the mechanical axis deviation was significantly changed with an average of 13.4 mm, which corresponded to 18% of the knee-joint width of the patients [22]. Wang et al. reported that leg alignment changed significantly depending on the position of the patient. Preoperatively, the mean HKAAs on double-leg standing and supine radiographs were 8° varus and 6.6° varus, respectively, while they were not significantly different postoperatively [23]. In our patients, the mean correction angle difference was approximately 2°, although only 2° appeared to be negligible. The regression analysis suggested that it may cause a 10% difference in %WBL. In addition, the range of the difference could be larger, especially in patients with a large alignment difference between the supine and standing conditions. In OWHTO, the alignment can be controlled by adjusting the amount of the gap and checking the alignment under fluoroscopy. However, checking the alignment using a rod may be inaccurate, and surgeons cannot be confident that the actual gap is the same as the estimated gap in the preoperative planning. Meanwhile, in CWHTO, the amount of bone resection is the most essential factor for determining the postoperative alignment. Therefore, reliable preoperative planning is necessary for obtaining the optimal target alignment during and after surgery. Considering that no statistically significant difference was found postoperatively between the supine and standing conditions, similar to Wang’s report, the alignment in the supine position during the surgery could be used at least to avoid large postsurgical correction errors.

The large difference between the supine and standing positions in some patients is presumably caused by increased medial joint laxity. Recent studies have revealed that preoperative varus soft-tissue laxity could cause overcorrection after HTO. Ogawa et al. reported that preoperative varus joint laxity determined by varus stress correlated with an increased correction angle after surgery [18]. So et al. examined the discrepancy in mechanical axis change between the values obtained during surgery by using a navigation system when postoperative standing radiographs were 2.0 ± 2.4° and reported that the difference in JLCA between the standing and supine radiographs was the most predictive factor for a correction error of > 3° [27]. Similarly, Lee et al. reported that a larger preoperative JLCA was associated with overcorrection after OWHTO [19]. Furthermore, Park et al. reported that overcorrection of > 10% difference in WBL was observed in 28% of the patients after OWHTO, and increased JLCA and valgus stress were risk factors for overcorrection [20]. In our study, the mean %WBLd was approximately 10%, and a %WBLd of > 10% was observed in 45% of patients. This suggests that a large overcorrection occurs frequently during surgery when HTO is performed according to a preoperative plan based on standing radiographs, as patients are placed in supine positions during the surgery. Therefore, to avoid overcorrection after HTO, it would be safer to use images taken in the supine position, particularly in patients with a large JLCA.

The achievement of optimal limb alignment after HTO has been controversial. Previous studies attempted to estimate the postoperative limb alignment based on the JLCA change using a lateral-wedge insole, preoperative stress radiography, and intraoperative stress test [18, 28–30]. However, these methods require strict control of the conditions such as weight-bearing position, magnitude and direction of forces, and a small difference in any one condition can cause a large difference in the alignment. By contrast, methods using supine radiographs appear to be simple. Ogata et al. previously examined the changes in the condylar-plateau angle, which corresponds to JLCA, before and after CWHTO [24]. In their study, a significant difference in condylar-plateau angle, which corresponds to JLCA, was found between the standing and supine radiographs preoperatively, and the mean condylar-plateau angle on the supine radiographs prior to surgery was similar to that on the standing radiographs after HTO. Therefore, the authors recommended the use of supine radiographs in the preoperative planning for HTO because the use of radiographs taken in standing positions could result in an unpredictable alignment correction. Furthermore, Shin et al. recently reported that preoperative planning using supine radiographs was a more predictive and accurate method for obtaining the expected limb alignment after OWHTO than that using standing radiographs [31]. These studies suggest that supine radiographs are more predictable than standing radiographs in their ability to achieve the desired alignment.

In this study, we found a weak to moderate correlation between the correction angle difference and the JLCA and FTA differences. Therefore, the JLCA or FTA can be used to adjust the correction angle or minimize the possibility of correction error by subtracting the angle difference from the estimated correction angle determined on standing radiographs. In some hospitals, it is not possible to perform whole-leg-view radiography in the supine position. In such cases, it is advisable to perform radiography including only the knee in the supine position and to measure the difference in the JLCA between the standing and supine conditions to adjust for the correction angle, especially in patients with a high JLCA on standing radiographs. In the present study, a linear correlation between the %WBLd and the correction angle difference was observed using the formula:

$$ \left(\%\mathrm{WBLd}=4.72\times \mathrm{correction}\ \mathrm{angle}\ \mathrm{difference}+0.08\right), $$

where %WBLD is the weight-bearing line discrepancy.

This linear correlation suggests that a 1° difference in the correction angle can result in a 5% difference in the %WBL. The formula may be useful to estimate the possible correction discrepancy during surgeries in the preoperative planning for HTO. Although limb alignment during surgery can be different from the postoperative limb alignment assessed in standing conditions, intraoperative assessment is still critical for surgeons. Jang et al. reported a significant linear relationship between the intraoperative post-osteotomy %WBL and the postoperative %WBL in the standing position after OWHTO. The results suggested that the postoperative standing %WBL could be estimated based on the assessment of %WBL during surgery [32]. Therefore, whole step-by-step meticulous analyses, including preoperative planning, assessment of parameters, and intraoperative alignment control, could reduce correction errors after the HTO. In the present study, %WBL differences were within 5% after surgery in most of the patients (total 87.5% of the patients), suggesting that supine radiographs during surgery could be used as an indicator for postoperative limb alignment. Since there was no obvious tendency toward an increase or a decrease in %WBL between standing and supine radiographs after surgery, the cause of the discrepancy of more than 5% is currently unknown. Further analyses are required.

The strength of this study lies in a fact that the position of a patient could affect the preoperative planning in HTO and the possible advantages of using supine radiographs were presented more clearly than in previous studies.

However, this study has some limitations. First, because analyses of postoperative alignment were not comprehensively included in this study, the actual alignments after surgery may have been different from the planned alignments. Second, although the %WBL determined using radiographs taken in the supine position was assumed to be the alignment during surgery, the alignment during surgery may have been different from the optimal alignment in the standing position. Third, stress radiographs were not used in this study. Although some surgeons recommend using stress radiographs to estimate and eliminate the effects of medial soft-tissue laxity on postoperative alignment, the amount of force that should be applied has not yet been optimized. Fourth, in the postoperative evaluation, surgical procedures were not entirely consistent. In addition, the alignment was not strictly checked during surgeries in all the analyzed patients. Fifth, the correction error between the actual planning and after surgery was not examined in this study. Therefore, more detailed analyses are required to demonstrate the advantages of using supine radiographs for HTO planning.

## Conclusions

We found significant differences in the estimated correction angles between the supine and standing radiographs in the planning for HTO, while no significant difference was found postoperatively. Therefore, surgeons should consider the difference between supine and standing radiographs and estimate the possible surgical correction error during HTO planning.

## Data Availability

The data and materials that support the findings of this study are available upon request.
